# Risk drinking levels and sex are associated with cancer and liver, respiratory, and other medical conditions

**DOI:** 10.1016/j.dadr.2021.100007

**Published:** 2021-11-26

**Authors:** Terril L. Verplaetse, Walter Roberts, MacKenzie R. Peltier, Yasmin Zakiniaeiz, Catherine Burke, Kelly E. Moore, Brian Pittman, Sherry A. McKee

**Affiliations:** a Department of Psychiatry, Yale School of Medicine, New Haven, CT, USA; b Psychology Service, Veterans Affairs Connecticut Healthcare System, West Haven, CT, USA; c Department of Psychology, East Tennessee State University, Johnson City, TN, USA

**Keywords:** Alcohol, Risk drinking, Sex, Gender, Medical conditions, Cancer

## Abstract

**Background:**

Heavy alcohol use is associated with increased risk of alcohol-related health consequences. Alcohol consumption has increased in females in the last fifteen years and females are more likely to experience exacerbated health risks due to drinking. Our group identified that females with AUD were more likely to report respiratory conditions or cancers compared to their male counterparts. This analysis sought to further examine relationships between sex and alcohol use on medical conditions by using the new 2020 U.S. Dietary Guidelines risk drinking levels.

**Methods:**

Data from the U.S. National Epidemiologic Survey on Alcohol and Related Conditions (NESARC-III; *n* = 36,309) was used to evaluate associations between sex (female vs. male) and alcohol risk drinking levels (abstainer, binge, heavy, extreme binge vs. moderate drinking) on past year self-reported doctor-confirmed medical conditions).

**Results:**

Females were 1.5 to 2 times more likely to have pain, respiratory, or other medical conditions in the past year (odds ratio [*OR*]=1.46–2.11) vs. males. Significant interactions demonstrated that heavy drinking females or extreme binge drinking females were 2 to 3 times more likely to have cancers or other conditions (*OR*=1.95–2.69) vs. males at the same risk drinking level. Female abstainers were more likely than male abstainers to have other medical conditions (*OR*=1.77).

**Conclusions:**

Consistent with our previous findings, results identify that higher risk drinking levels are associated with the presence of past year self-reported doctor-confirmed medical conditions spanning organ systems, particularly in females. Treatment for high-risk drinking should be considered in the clinical care of individuals with significant medical conditions.

## Highlights

• We examined sex by risk drinking levels on past year medical conditions with NESARC.
• Females had greater odds of pain and respiratory conditions vs. males.
• Abstainers were more likely to have some medical conditions vs. moderate drinkers.
• Heavy drinking females were more likely to have cancers vs. heavy drinking males.
• Higher risk drinking may be associated with conditions spanning organ systems.

## 1 Introduction

Alcohol is a significant public health burden, with robust increases in alcohol-related harms and mortality in the U.S. in the last two decades (White et al., 2020). The Centers for Disease Control and Prevention (CDC) found an average of 93,296 alcohol-attributable deaths per year and 2.7 million years of potential life lost in the U.S. (Esser et al., 2020). Others found that alcohol-related deaths in the U.S. doubled between 1999 and 2017 (White et al., 2020). More than 55% of alcohol-related deaths were caused by chronic conditions, such as alcoholic liver disease or liver cirrhosis and cardiovascular diseases (Esser et al., 2020). These results are consistent with a larger body of literature (e.g., case-control, clinical or experimental, and cohort studies) identifying that those engaging in heavy alcohol use are at increased risk for health consequences related to their drinking (Rehm et al., 2010; Szabo, 2018).

There has been a substantial increase in rates of alcohol use over the last decade, primarily in women (Grucza et al., 2018; Grant et al., 2017). A recent meta-analysis of six national surveys found that increases in drinking and binge drinking over the last fifteen years were driven solely by females (Grucza et al., 2018). Research also demonstrates that females experience worse alcohol-related health consequences compared to males, including alcohol-related cancers, stroke among women consuming 3 or more drinks per day, brain damage, and sex-specific consequences such as spontaneous abortion, perinatal mortality, and menstrual irregularity (National Institute of Alcohol Abuse and Alcoholism, 2019). Because women are consuming more alcohol and are more likely to experience exacerbated health risks due to drinking, it is critical to identify associations between alcohol use and health conditions in females compared to males for effective treatment interventions.

Previous findings from our group identified that females with an ongoing Diagnostic and Statistical Manual of Mental Disorders Fifth Edition (DSM-5) alcohol use disorder (AUD) were 2 to 3 times more likely to report doctor- or health professional-confirmed respiratory conditions or cancers compared to males with AUD (Verplaetse et al., 2021). Thus, DSM-5 AUD diagnoses may be related to the presence of health conditions across organ systems, especially in females. However, to our knowledge, the extent to which these findings are specific to individuals with AUD or extend to risk drinking levels, designated by the U.S. Dietary Guidelines and ranging from alcohol abstinence and moderate drinking to heavy and extreme binge drinking, have not been established. That is, is an AUD diagnosis necessary for significant associations with the presence of other past year medical conditions or are varying levels of alcohol consumption, not necessarily AUD, also associated with increased odds of past year medical conditions in females and males?

The aim of this analysis was to examine whether progressively increasing risk drinking levels, set forth by the new 2020 U.S. Dietary Guidelines (United States Department of Agriculture, 2020), and sex were associated with doctor- or health professional-confirmed medical disease categories in a nationally representative sample of U.S. adults (NESARC-III), including conditions affecting the liver, cardiovascular and respiratory systems, cancers, and pain. We hypothesized that higher risk drinking categories or progressively increasing cut-points of alcohol use (i.e., heavy drinking, extreme binge drinking) would be associated with past year medical conditions spanning organ systems, and that this association would be stronger in females. It is important to note that causal or temporal relationships between risk drinking levels and past year medical conditions cannot be addressed in the present investigation due to the cross-sectional nature of the NESARC.

## 2 Materials and methods

### 2.1 Data source

The NESARC-III data was used for this study. NESARC-III (2012–2013) was carried out by the National Institute on Alcohol Abuse and Alcoholism (NIAAA). The sample consisted of *n* = 36,309 non-institutionalized adults living in the United States. All individuals who agreed to participate completed an in-person computer-assisted interview consisting of the NIAAA Alcohol Use Disorder and Associated Disabilities Interview Schedule-5 (AUDADIS-5) and other questions pertaining to alcohol use. Hispanic, African American, and Asian respondents were oversampled. Data were adjusted for oversampling and non-response, then weighted to represent the U.S. civilian population. Methodology used in the NESARC-III survey are detailed elsewhere (Grant et al., 2014; Dawson et al., 2015).

### 2.2 Sex

The NESARC-III did not ask respondents their sex *except* in instances where the interviewer could not make a reasonable determination based on the way the respondent presented themselves. If not apparent, NESARC-III asked “What is your sex?” and recorded each respondent's sex as either male or female. Gender identity was not queried.

### 2.3 Risk drinking levels

Risk drinking levels followed the 2020 U.S. Dietary Guidelines for alcohol use, and we included heavy drinking and extreme binge drinking. We coded the NESARC-III data into the following categories: *abstainer*, former drinker or lifetime abstainer; *moderate*, ≤2 drinks/day for males and ≤1 drink/day for females; *binge*, 5+ drinks/2 h for males and 4+ drinks/2 h for females; *heavy*, 4+ drinks/day for males and 3+ drinks/day for females; *extreme binge*, 8+ drinks/day for females and 12+ drinks/day for males. NESARC-III queried participants on quantity of alcohol consumption on days when drank in the last 12 months.

### 2.4 Medical conditions

The NESARC recorded the presence or absence of 32 medical conditions in the *last 12 months* and whether the diagnosis was confirmed by a doctor or other health professional. For the present investigation, we used doctor- or health professional-confirmed diagnoses for each medical condition, except for HIV/AIDS. For HIV/AIDS, respondents were asked if they *ever* tested positive for HIV or AIDS? NESARC-III did not ask whether HIV/AIDS status was confirmed by a doctor or health professional. The NESARC questions regarding whether health conditions were diagnosed by a doctor or other health professional were self-reported by respondents. Medical conditions spanned liver, cardiovascular, and respiratory conditions, cancers, pain disorders, seizure disorders, HIV/AIDS, insomnia, bowel problems, sexually transmitted diseases, traumatic brain injuries, etc. A complete list of medical conditions from NESARC-III by category can be found in Table 1.

**Table 1:** List of medical conditions^1^ within each broad medical condition category.

n (%)
*Liver*
1. Cirrhosis of the liver
2. Other form of liver disease
*Cardiovascular*
3. Hardening of the arteries or arteriosclerosis
4. High blood pressure or hypertension
5. High cholesterol
6. High triglycerides
7. Chest pain or angina
8. Rapid heartbeat or tachycardia
9. A heart attack or myocardial infarction
10. Other form of heart condition or heart disease
11. Stroke
*Cancer*
12. Breast cancer
13. Cancer of the mouth, tongue, throat, or esophagus
14. Liver cancer
15. Other cancer
*Pain*
16. Arthritis
17. RSD or CRPS
18. Fibromyalgia
19. Other nerve problem in the legs, arms, or back
*Respiratory*
20. Lung problems
21. Tuberculosis
*Other*
22. Diabetes
23. Stomach ulcer
24. STD
25. Epilepsy or seizure disorder
26. Problems falling asleep or staying asleep
27. Anemia
28. Bowel problems
29. Osteoporosis
30. Pancreatitis
31. Traumatic brain injury
32. HIV/AIDS?

Note: ^1^For the present investigation, we used doctor or health professional confirmed diagnoses for each medical condition, except for HIV/AIDS. For HIV/AIDS, respondents were asked if they *ever* tested positive for HIV or AIDS? NESARC-III did not ask whether HIV/AIDS status was confirmed by a doctor or health professional. NESARC-III recorded the presence of all other medical conditions in the *last 12 months*. NESARC, National Epidemiologic Survey on Alcohol and Related Conditions-III; RSD, reflex sympathetic dystrophy; CRPS, Complex Regional Pain Syndrome; STD, sexually transmitted disease; HIV, human immunodeficiency virus; AIDS, acquired immunodeficiency syndrome.

### 2.5 Statistical analysis

Data were analyzed using PROC SURVEYLOGISTIC in SAS, version 9.4 (SAS v9.4, SAS Institute Inc., Cary, NC), which allowed for incorporating the stratification, clustering (i.e., primary sampling unit (PSU)), and unequal weighting of the sampling design. Binary logistic regression modeling was used to examine associations between sex (female vs. male) and risk drinking levels (abstainer, binge drinking, heavy drinking, extreme binge drinking vs. moderate drinking) with the presence of medical conditions (yes vs. no) by broad disease category: liver, cardiovascular, cancer, pain, respiratory, and other. Due to small cell sizes, additional models with individual medical conditions within each category as dependent variables could not be analyzed. The main effect of each variable of interest on any given outcome was interpreted relative to our chosen reference (i.e., male, moderate drinking). Covariates relevant to health outcomes, including age, race/ethnicity, body mass index (BMI), smoking status, other drug use, and income, were included in the analysis. Relationships between sex and risk drinking levels were assessed in terms of odds ratios and were considered significant at *p* ≤ 0.05.

## 3 Results

Sample characteristics by sex are summarized in Table 2. As in the first manuscript (Verplaetse et al., 2021), all chi-square analyses performed to examine sex differences in sample characteristics were significant at *p*<0.001, except age (*p* = 0.07). Males had higher rates of moderate (39.8% vs. 32.8%), binge (4.3% vs. 2.8%), and extreme binge (15.2% vs. 9.0%) drinking compared to females. More females reported alcohol abstinence (39.6% vs. 26.9%) and heavy drinking (15.8% vs. 13.9%) compared to males (see Table 2).

**Table 2:** Sample characteristics by sex (NESARC-III, *n* = 36,309).

		Male	Female	χ^2^	*p*
Risk Drinking Level (%)				797.55	<0.001
	Abstainer	26.9	39.6		
	Moderate	39.8	32.8		
	Binge	4.3	2.8		
	Heavy	13.9	15.8		
	Extreme binge	15.2	9.0		
Age (%)				5.45	0.07
	18–29	22.9	21.9		
	30–44	27.5	28.2		
	45+	49.5	49.9		
Race/ethnicity (%)				47.08	<0.001
	White	53.9	52.0		
	Black	19.9	22.6		
	Native American	1.3	1.5		
	Asian	5.4	4.6		
	Hispanic	19.5	19.3		
BMI (%)				455.52	<0.001
	<18.5	0.8	2.0		
	18.5 - 24.9	29.7	35.5		
	25.0 - 29.9	39.3	28.7		
	≥30.0	30.1	33.8		
Smoking status (%)				1020.29	<0.001
	Current smoker	34.6	22.3		
	Former smoker	18.7	14.5		
	Non-smoker	46.7	63.2		
Drug use (%)				489.48	<0.001
	Drug non-user	57.7	69.0		
	Drug user	42.3	31.0		
Income (%)				304.42	<0.001
	$9,999 or less	8.7	10.9		
	$10,000 - $24,999	22.2	27.8		
	$25,000 - $49,999	27.7	27.7		
	$50,000 or higher	41.4	33.6		

Note: NESARC, National Epidemiologic Survey on Alcohol and Related Conditions-III; DSM, Diagnostic and Statistical Manual of Mental Disorders – Fifth Edition; BMI, body mass index. Drug user is defined as ever use of sedatives/tranquilizers, painkillers, marijuana, cocaine/crack, stimulants, club drugs, hallucinogens, inhalants, heroin, and other drugs (e.g., steroids) and drug non-user is defined as never use of the above-mentioned drugs.

### 3.1 Medical conditions

#### 3.1.1 Liver

Risk drinking levels were associated with past year liver conditions (*p* = 0.01). Alcohol abstainers were more likely to have a past year liver condition compared to moderate drinkers (*OR*=2.009, *95% CI*=1.434, 2.815). A significant interaction (*p* = 0.003) between sex and risk drinking levels demonstrated that female abstainers were less likely to have past year liver conditions compared to male abstainers (*OR*=0.519, *95% CI*=0.341, 0.791; see Table 3).

**Table 3:** Associations of sex and risk drinking levels with the presence of past year medical condition categories.

	**Sex** (female vs. male)						**Risk Drinking Level** (moderate vs. all other levels)					**Sex by Risk Drinking Level**	
												(F v. M by risk drinking level)	
	OR (95% CI)		*p*			Overall *p*	OR (95% CI)	*p*		Overall *p*		OR (95% CI)	*p*
													
**Liver**	0.803 (0.561, 1.150)		0.229			**0.001**				**0.039**			
					Abstainer		**2.009 (1.434, 2.815)**	**<0.0001**			Moderate	1.197 (0.749, 1.913)	0.450
					Binge		1.440 (0.594, 3.489)	0.416			Abstainer	**0.519 (0.341, 0.791)**	**0.003**
					Heavy		1.145 (0.709, 1.849)	0.577			Binge	0.572 (0.115, 2.842)	0.491
					Extreme binge		1.252 (0.804, 1.948)	0.317			Heavy	0.737 (0.366, 1.481)	0.388
											Extreme binge	1.276 (0.693, 2.353)	0.431
**Cardiovascular**	0.923 (0.832, 1.024)		0.130			0.386				0.067			
					Abstainer		1.007 (0.916, 1.106)	0.890			Moderate	0.917 (0.813, 1.035)	0.158
					Binge		0.927 (0.758, 1.134)	0.459			Abstainer	1.113 (0.985, 1.258)	0.085
					Heavy		0.910 (0.817, 1.014)	0.087			Binge	0.786 (0.532, 1.159)	0.221
					Extreme binge		0.946 (0.841, 1.064)	0.350			Heavy	0.845 (0.701, 1.019)	0.077
											Extreme binge	0.989 (0.792, 1.235)	0.923
**Cancer**	1.217 (0.926, 1.600)		0.157			0.516				**0.003**			
					Abstainer		1.080 (0.915, 1.274)	0.359			Moderate	0.952 (0.747, 1.214)	0.692
					Binge		1.162 (0.687, 1.965)	0.572			Abstainer	0.808 (0.634, 1.031)	0.085
					Heavy		0.943 (0.691, 1.288)	0.709			Binge	0.785 (0.295, 2.089)	0.625
					Extreme binge		0.818 (0.543, 1.232)	0.333			Heavy	**1.945 (1.116, 3.391)**	**0.019**
											Extreme binge	**2.275 (1.024, 5.055)**	**0.044**
**Pain**	**1.459 (1.304, 1.631)**		**<0.0001**			**0.013**				0.379			
					Abstainer		1.051 (0.953, 1.158)	0.319			Moderate	1.609 (1.446, 1.790)	<0.0001
					Binge		**0.794 (0.637, 0.990)**	**0.041**			Abstainer	1.593 (1.384, 1.833)	<0.0001
					Heavy		0.896 (0.776, 1.035)	0.134			Binge	1.308 (0.801, 2.136)	0.281
					Extreme binge		**0.814 (0.710, 0.933)**	**0.004**			Heavy	1.575 (1.288, 1.926)	<0.0001
											Extreme binge	1.250 (0.974, 1.605)	0.079
**Respiratory**	**1.635 (1.307, 2.046)**		**<0.0001**			**<0.0001**				0.273			
					Abstainer		**1.550 (1.331, 1.805)**	**<0.0001**			Moderate	1.292 (0.997, 1.675)	0.053
					Binge		1.038 (0.667, 1.616)	0.868			Abstainer	1.441 (1.183, 1.754)	0.0004
					Heavy		1.057 (0.847, 1.320)	0.622			Binge	1.875 (0.823, 4.271)	0.133
					Extreme binge		1.183 (0.926, 1.512)	0.176			Heavy	1.454 (1.004, 2.107)	0.048
											Extreme binge	2.303 (1.517. 3.495)	0.0001
**Other**	**2.113 (1.900, 2.349)**		**<0.0001**			**<0.0001**				**0.030**			
					Abstainer		**1.245 (1.145, 1.355)**	**<0.0001**			Moderate	**1.799 (1.592, 2.033)**	**<0.0001**
					Binge		**0.803 (0.657, 0.982)**	**0.033**			Abstainer	**1.768 (1.576, 1.983)**	**<0.0001**
					Heavy		**0.832 (0.732, 0.946)**	**0.005**			Binge	**2.217 (1.436, 3.425)**	**0.0004**
					Extreme binge		0.950 (0.853, 1.058)	0.344			Heavy	**2.219 (1.822, 2.704)**	**<0.0001**
											Extreme binge	**2.689 (2.108, 3.430)**	**<0.0001**

Note: bold typeface, *p* ≤ 0.05; OR, odds ratio; CI, confidence interval; F, female; M, male; AUD, alcohol use disorder.

We conducted exploratory analyses to examine whether this effect was due to abstainers being former drinkers and possibly abstaining as a results of liver disease. Results did not change when drinking status (current, former, lifetime abstainer) was included as a covariate in the analysis.

#### 3.1.2 Cardiovascular

Main effects of sex and risk drinking levels as well as the interaction between sex and risk drinking levels were not significant (*p*>0.05; see Table 3).

#### 3.1.3 Cancer

A significant interaction (*p* = 0.003) between sex and risk drinking levels demonstrated that heavy drinking females were more likely to have past year cancers compared to heavy drinking males (*OR*=1.945, *95% CI*=1.116, 3.391). Further, females who extreme binge drank in the last year were more likely to have past year cancers compared to extreme binge drinking males (*OR*=2.275, *95% CI*=1.024, 5.055; see Table 3).

#### 3.1.4 Pain

Sex (*p*<0.0001) and risk drinking levels (*p* = 0.013) were associated with past year pain conditions. Females were more likely than males to have past year pain conditions (*OR*=1.459, *95% CI*=1.304, 1.631). Binge drinkers and extreme binge drinkers were less likely than moderate drinkers to have past year pain conditions (*OR*=0.794, *95% CI*=0.637, 0.990 and *OR*=0.814, *95% CI*=0.710, 0.933, respectively; see Table 3).

#### 3.1.5 Respiratory

Sex (*p*<0.0001) and risk drinking levels (*p*<0.0001) were associated with past year respiratory conditions. Females were more likely than males to have past year respiratory conditions (*OR*=1.635, *95% CI*=1.307, 2.046). Abstainers were more likely than moderate drinkers to have past year pain conditions (*OR*=1.550, *95% CI*=1.331, 1.805; see Table 3).

#### 3.1.6 Other

Sex (*p*<0.0001) and risk drinking levels (*p*<0.0001) were associated with the presence of other conditions in the past year. Females were more likely to have other conditions in the past year compared to males (*OR*=2.113, *95% CI*=1.900, 2.349). Abstainers were more likely to have other conditions in the past year compared to moderate drinkers (*OR*=1.245, *95% CI*=1.145, 1.335). Binge drinkers and heavy drinkers were less likely to have other conditions in the past year compared to moderate drinkers (*OR*=0.803, *95% CI*=0.657, 0.982 and *OR*=0.832, *95% CI*=0.732, 0.946, respectively). A significant interaction (*p* = 0.030) between sex and risk drinking levels demonstrated that females were more likely to have other medical conditions across all risk drinking levels. Female abstainers were more likely than male abstainers to have past year other conditions (*OR*=1.768 *95% CI*=1.576, 1.983). Further, moderate drinking females and binge drinking females were more likely to have past year other conditions compared to males drinking at the same levels (*OR*=1.799, *95% CI*=1.592, 2.033 and *OR*=2.217, *95% CI*=1.436, 3.425, respectively). Heavy drinking females and females who extreme binge drank had higher odds of having other conditions in the past year compared to heavy drinking males and extreme binge drinking males (*OR*=2.219, *95% CI*=1.822, 2.704 and *OR*=2.689, *95% CI*=2.108, 3.430, respectively; see Table 3).

## 4 Discussion

To our knowledge, this investigation was the first to examine relationships between sex, risk drinking levels set forth by the 2020 U.S. Dietary Guidelines, and the presence of liver, cardiovascular, cancer, pain, respiratory, and other medical conditions in a large cross-sectional dataset of nationally representative U.S. adults (NESARC-III). Females who engaged in heavy drinking or extreme binge drinking in the past year were 2 to 3 times more likely to have a past year doctor- or health-professional confirmed medical condition compared to males engaging in heavy drinking or extreme binge drinking, respectively. Main effects of risk drinking levels demonstrated that alcohol abstainers were 1.5 to 2 times more likely to have liver, respiratory, or other medical conditions in the past year compared to moderate drinkers. When examining main effects of sex, females were 1.5 to 2 times more likely to have pain, respiratory, and other medical conditions compared to males.

Results suggest that problematic drinking may be related to negative health outcomes, especially in females. This is consistent with findings from our group and others demonstrating that females have higher odds of alcohol-related health consequences relative to males (Verplaetse et al., 2021; Erol and Karpyak, 2015; Szabo, 2018; National Institute of Alcohol Abuse and Alcoholism, 2019), even though females may consume less alcohol and/or use alcohol for a shorter time than males (Rehm et al., 2010; Peltier et al., 2019). Results from our first manuscript found that females with ongoing AUD were 2 to 3 times more likely than males to have respiratory conditions and cancers (Verplaetse et al., 2021). The present investigation found that females engaging in problematic drinking (e.g., binge, heavy, extreme binge) were 2 to 3 times more likely than males to have cancers and other significant medical conditions. This overlap suggests that both AUD and progressively risky drinking are robustly associated with cancers in females. Tailored treatments for cancers and other medical conditions should consider frequency of alcohol use, especially in females.

Worth noting, results may reflect the increased likelihood of females to seek medical care for health problems. Research suggests that males are less likely to seek medical help compared to their female counterparts (Powell et al., 2016; Vaidya et al., 2012). Treatment-seeking bias must be considered when interpreting results from the present investigation. Thus, results may merely suggest that females are more likely to seek care from a doctor or health professional rather than an association between increasing cut-points of alcohol use and the presence of health conditions in females versus males.

In the present investigation, individuals engaging in binge drinking or extreme binge drinking had 18.6 – 20.6% lower odds of past year pain disorders. While we cannot identify causal or temporal relationships in the current study, previous work suggests a link between chronic pain and alcohol use. Alcohol has analgesic properties; thus, alcohol use may be used as a form of self-medication (Horn-Hofmann et al., 2015). Extreme binge drinking may be used as a coping mechanism so individuals engaging in this behavior may be less likely to report pain. However, other studies suggest that chronic alcohol consumption may exacerbate chronic pain disorders (Apkarian et al., 2013). Problematic drinking was not associated with any other medical condition categories except 'other' significant medical conditions, and this may be related to small risk drinking level by medical condition cell sizes.

It should be noted that alcohol abstinence was associated with increased odds of liver, respiratory, and other medical conditions compared to moderate drinking. This effect also extended to a significant interaction between sex and risk drinking levels, such that females abstaining from alcohol were 2 times more likely to have other significant medical conditions compared to males. Thus, moderate alcohol consumption may be associated with less health consequences than alcohol abstinence. Some research has pointed to beneficial effects of moderate drinking on health and mortality, including protective effects on Alzheimer's disease and cardiovascular health (Ronksley et al., 2011; Di Castelnuovo et al., 2006; Gaziano et al., 2000; Baum-Baicker, 1985; Fernandez-Sola, 2015; Piazza-Gardner et al., 2013). Nonetheless, work suggesting health benefits of moderate alcohol consumption remains inconclusive or even controversial (Burton and Sheron, 2018; Stockwell et al., 2016; Piazza-Gardner et al., 2013). It may be possible that individuals who are sicker are taking more medications that are contraindicated with alcohol and thus cannot drink or were diagnosed with medical conditions some time ago and, as a result, are now abstaining from alcohol. Thus, multiple factors may be related to this finding and alcohol may not necessarily be protective of liver, respiratory, or other health conditions.

### 4.1 Limitations

Limitations are discussed in detail in the first manuscript (Verplaetse et al., 2021). In brief, due to the cross-sectional nature of the NESARC, causal or temporal relationships in the current investigation cannot be addressed between risk drinking levels and medical conditions. Future work should examine directionality of the relationship between risk drinking levels and diagnosed medical conditions. Second, the NESARC-III asked questions regarding the presence of medical conditions in the past 12 months only and does not account for diagnoses outside of this period. Relatedly, respondents were not asked about specific instances of inpatient hospitalizations, emergency department visits, outpatient office visits, or clinic visits and no collateral information about medical conditions was obtained beyond self-report of a doctor's diagnosis. Fourth, the NESARC-III did not distinguish between former drinkers and lifetime abstainers when asking about quantity or frequency of alcohol use. Thus, former drinkers and lifetime abstainers were categorized as abstainers in the present study. However, those who are now sober (i.e., former drinkers) may have experienced medical problems that contributed to their decision to no longer drink. This should be explored in future studies. Finally, as previously mentioned, cell sizes were relatively small (e.g., *n*<50) for some sex by risk drinking level by medical condition categories. Future work should seek to examine these relationships in larger samples of drinkers across risk drinking levels and doctor-confirmed medical diagnoses beyond self-report.

### 4.2 Conclusion

This report examined relationships between sex, the new 2020 U.S. Dietary Guidelines risk drinking levels, and doctor- or health professional-confirmed medical disease categories in a nationally representative sample of U.S. adults. To our knowledge, this is the first investigation to examine such relationships using NESARC-III. Results suggest that females as well as alcohol abstainers may be more likely to have a past year doctor- or health professional-confirmed medical diagnoses, such as liver, pain, and other medical conditions compared to males and moderate drinkers, respectively. Significant interactions identified that females who drank heavily or engaged in extreme binge drinking were 2 – 3 times more likely to have cancers or other medical conditions compared to males with similar drinking habits. It is important to note that findings do not imply causal or temporal relationships; however, results are largely consistent with findings from our first manuscript examining associations between sex and ongoing AUD on cancers and respiratory and other medical conditions (Verplaetse et al., 2021) and with other work indicating that chronic problematic drinking plays a role in worsened health outcomes, especially in women.

## Contributors

Terril Verplaetse and Sherry McKee conceptualized the study. All authors contributed to study design. Terril Verplaetse and Brian Pittman conducted the statistical analysis. Terril Verplaetse wrote the first draft of the manuscript and all authors contributed to and have approved the final manuscript.

## Funding

This work was supported by 10.13039/100000027NIAAA grants K01AA025670 (TLV), R03AA028361 (TLV), K23AA026890 (WR), P01AA027473 (SAM), and U54AA027989 (SAM).

## Declaration of Conflicting Interests

All authors declare that they have no conflicts of interest.
